# Mechanism of Molecular Activity of Yolkin—a Polypeptide Complex Derived from Hen Egg Yolk—in PC12 Cells and Immortalized Hippocampal Precursor Cells H19-7

**DOI:** 10.1007/s12035-023-03246-6

**Published:** 2023-02-03

**Authors:** Wioletta Kazana, Dominika Jakubczyk, Jakub Siednienko, Aleksandra Zambrowicz, Józefa Macała, Agnieszka Zabłocka

**Affiliations:** 1 Lab. Microbiome Immunobiology, Hirszfeld Institute of Immunology and Experimental Therapy, Polish Academy of Sciences, R. Weigla 12, 53-114 Wrocław, Poland; 2 Bioengineering Research Group, Łukasiewicz Research Network-PORT Polish Center for Technology Development, 54-066 Wroclaw, Poland; 3 Department of Functional Food Products Development, Faculty of Biotechnology and Food Sciences, Wrocław, University of Environmental and Life Sciences, Chełmońskiego 37, 51-630 Wrocław, Poland

**Keywords:** Yolkin, Aging, Brain-derived neurotrophic factor, Carboxypeptidase E, CREB transcription factor

## Abstract

Food-derived bioactive peptides able to regulate neuronal function have been intensively searched and studied for their potential therapeutic application. Our previous study showed that a polypeptide complex yolkin, isolated from hen egg yolk as a fraction accompanying immunoglobulin Y (IgY), improved memory and cognitive functions in rats. However, the mechanism activated by the yolkin is not explained. The goal of the present study was to examine what molecular mechanism regulating brain-derived neurotrophic factor (BDNF) expression is activated by the yolkin complex, using in vitro models of PC12 cell line and fetal rat hippocampal cell line H19-7. It was shown that yolkin increased the proliferative activity of rat hippocampal precursor cells H19-7 cells and upregulated the expression/production of BDNF in a cyclic adenosine monophosphate (cAMP)-response element-binding protein (CREB)-dependent manner. Additionally the upregulation of carboxypeptidase E/neurotrophic factor–α1 (CPE/(NF-α1) expression was shown. It was also determined that upregulation of CREB phosphorylation by yolkin is dependent on cyclic adenosine monophosphate/protein kinase A (cAMP/PKA) and phosphoinositide 3-kinases/protein kinase B (PI3K/Akt) signaling pathway activation. Moreover, the impact of yolkin on the level of intracellular Ca^2+^, nitric oxide, and activation of extracellular signal-regulated kinases 1/2 (ERK 1/2 kinase) was excluded. These results emphasize that yolkin can act comprehensively and in many directions and may participate in the regulation of neurons’ survival and activity. Therefore, it seems that the yolkin specimen can be used in the future as a safe, bioavailable, natural nutraceutical helping to improve the cognition of older people.

## Introduction

Aging is a natural process, and its consequences are ranging from physical to behavioral aspects [1, 2]. It is a progressive deterioration of the homeostatic brain mechanisms, accompanied by cognitive decline. Our brains, with time, become more susceptible to learning and memory impairments, generally attributed to a decrease in neuronal plasticity of the cortex and hippocampus [3]. These regions are important for cognitive functions, memory and spatial learning tasks, and regulation of emotions. Studies by Erickson et al. revealed that shrinkage of the hippocampus may be strongly related to the decreased level of brain-derived neurotrophic factor (BDNF). Moreover, other studies proved that induction of neurotrophin production and secretion in the hippocampus of aged mice could rescue the long-term potentiation and relieve spatial memory deficits [4, 5].

BDNF, the main neurotrophin produced by neurons, is crucial in neuronal development, proliferation, survival, and control of learning, memory formation, and synaptic plasticity [6–9]. Apart from that, BDNF was able to protect neurons against apoptosis and improve cognitive impairment caused by toxic amyloid beta (Aβ) deposition [10]. Next to BDNF, a new neurotrophic factor–α1(NF-α1) involved in neuroprotection named carboxypeptidase E (CPE) was lastly discovered [11]. CPE can act as a pro-BDNF sorting receptor [12], regulates BDNF-TrkB signaling in the hippocampus [13], and controls learning and memory processes [14]. Transcription factors like cyclic adenosine monophosphate (cAMP)-response element-binding protein (CREB) and nuclear factor қB (NF-қB) participate in the regulation of expression of genes coding pro-survival proteins and neurotrophins as BDNF [15, 16]. Their activation can be triggered by an increased level of the intracellular Ca^2+^ ions resulting in Ca^2+^/calmodulin-dependent protein kinase II/IV (CAMKII/IV), cAMP production, or activation of tropomyosin receptor kinase (Trk) receptors leading to activation of kinases such as protein kinase A (PKA), ERKs, or PI3K/Akt [15]. The potential role of nitric oxide (NO) in the activation of guanylate cyclase/cyclic guanosine monophosphate (GC/cGMP)/CREB pathway was also demonstrated [17].

Recently, there has been a large increase in interest in naturally occurring bioactive substances demonstrating neuroprotective potential [18, 19]. Promising seems to be the substances isolated from natural resources possessing neuroprotective activity, which can be used as safe, effective, and long-term supplements [20]. Regular supplementation by the neuroprotective agents may be an important therapeutic aspect improving mental health and significantly reducing the risk of neurodegenerative disease development. Bioactive food-derived compounds, because of their neuroprotective potential, as well as the high safety profile, are relevant in the obtainment of natural drugs and nutraceuticals [21, 22]. Yolk and egg white are the natural, rich sources of biologically active substances [23]. The precursor of the major proteins in the egg yolk is vitellogenin, which during egg formation is enzymatically cleaved into fragments situated afterwards in yolk granules or plasma [24–26]. It was determined by Polanowski et al. [24, 25] that hen egg yolk immunoglobulin Y is accompanied by polypeptide complex, named yolkin, representing vitellogenin-derived peptides. Yolkin consists of several peptides of molecular weight ranging from 1 to 35 kDa. In this complex, polypeptides of the molecular weight from 16 to 23 kDa are prevailing. The results of the research obtained up to now have demonstrated that yolkin possesses an immunoregulatory, an antioxidant, and a neuroprotective activity. It stimulates human whole blood (ex vivo) and mouse macrophages to produce cytokines, reduces the level of intracellular free oxygen radicals and lipid peroxidation products, and modulates nitric oxide production by mouse macrophages [24–27].

Behavioral studies performed by Lemieszewska et al. [28] has shown that yolkin can improve the cognitive function in both young and old rats. It was observed that yolkin alleviates behavioral symptoms of aging and promotes cognitive learning and memory in both young and old rats. These observations prompted us to study what intracellular mechanism can be triggered by yolkin in neuronal cells and what neuroprotective proteins are expressed in response to their activation.

In the present study, we deciphered that yolkin stimulates both neuron-like PC12 cells and immortalized hippocampal precursor cells H19-7 to produce and release significant amounts of mature form of BDNF. This effect was related to the ability of yolkin to trigger of cAMP/PKA and PI3K/Akt-dependent CREB activation. Additionally, the impact of yolkin on upregulation of CPE/NF-α-1 expression was shown. The data presented in this paper shed some light on the potential molecular mechanisms activated in neurons in response to yolkin polypeptide complex.

## Materials and Methods

### Reagents and Chemicals

High-glucose Dulbecco’s modified Eagle’s medium (DMEM) and Opti-MEM/GlutaMAX were from Gibco (Thermo Scientific, Waltham, MA, USA)); phosphate-buffered saline (pH 7.4), L-glutamine, antibiotics, (penicillin/streptomycin mixture, G-418), donor horse serum, and fetal bovine serum (FBS) were from Biowest (Nuaille, France). 3-(4,5-dimethylthiazol-2-yl)-2–5-diphenyltetrazolium bromide (MTT) and adenosine (Ado) were purchased from Sigma (St. Louis, MO, USA). Reagents for SDS-PAGE were from Bio-Rad (California, USA). NGF (from mouse submaxillary glands) and BDNF Emax ImmunoAssay System were from Promega (Madison, USA). PageRuler™ Plus Prestained Protein Ladder (10–250 kDa) was obtained from Thermo Scientific (Waltham, MA, USA). Anti ERK 1/2, anti-phospho ERK 1/2, anti-CREB, anti-phospho-CREB, anti-Akt, anti-phospho-Akt, anti-NF-қB, anti-phospho-NF-қB, anti-β-actin monoclonal antibodies, and anti-rabbit IgG AP-linked antibody were obtained from Cell Signaling Technology (Leiden, The Netherlands). Anti-CPE monoclonal antibody was from Invitrogen (Massachusetts, USA). Anti-BDNF polyclonal antibody was from ABclonal (MA, USA). 5-Bromo4-chloro-3-indolyl phosphate disodium salt (BCIP) and nitro blue tetrazolium (NBT) were from Carl Roth GmbH (Karlsruhe, Germany). LY294002 was obtained from MedChemExpress (New York, USA).

### Cell Cultures

PC12 (Tet-On) cell line (ClonTech), a rat pheochromocytoma cell line was used as a TrkB(-) cellular model. The cells respond reversibly to NGF by the induction of the neuronal phenotype. PC12 cells were maintained in Dulbecco’s modified Eagle’s medium (DMEM) with 10% fetal bovine serum (FBS), 5% horse serum, and 100 units of penicillin–streptomycin (PS) at 37℃, 5% CO_2_ in a humidified incubator. The cells were grown at least 2 weeks after thawing; the medium was changed every 2–3 days. The cells were collected from the flask for the experiments when cells reached 80% confluency. Experiments were performed using cells maintained between passages 2 and 8.

H19-7 cell line (ATCC) was used as a TrkB ( +) model. The cells were originally derived from hippocampi dissected from embryonic day 17 (E17) Holtzman rat embryos and immortalized by retroviral transduction of temperature-sensitive tsA58 SV40 large T antigen [29, 30]. H19-7 can be differentiated to a neuronal phenotype at the nonpermissive temperature (39 °C) when induced by basic fibroblast growth factor (bFGF) in an N2 medium. Briefly, H19-7 cells were maintained in Dulbecco’s modified Eagle’s medium with 4 mM L-glutamine adjusted to contain 1.5 g/L sodium bicarbonate and 4.5 g/L glucose supplemented with 10% fetal bovine serum, 200 µg/ml G-418, and 100 units of penicillin–streptomycin (PS). The cells were incubated at 33 °C and with the supplement of 5% CO_2_. The cells were grown at least 2 weeks after thawing; the medium was changed every 2–3 days. The cells were collected from the flask for the experiments when cells reached 80% confluency. Experiments were performed using cells maintained between passages 2 and 8.

Both cell lines were a gift from Dr. Janusz Matuszyk from the Hirszfeld Institute of Immunology and Experimental Therapy PAS, Poland.

### Isolation of Yolkin Polypeptide Complex

Yolkin was isolated from hen egg yolks according to the procedure described in detail by Polanowski et al. [25]. Briefly, the water solution of IgY preparation was the starting material for the isolation of immunologically active peptides. The native IgY, isolated from hen egg yolk after being dialyzed for 2 days against two changes of 100 mM of potassium phosphate buffer, pH 7.2 and clarified by centrifugation, was chromatographed on a Sephacryl S-100 HR column (K50/100 Pharmacia Ltd, Kent, UK) equilibrated with the same buffer. Fractions, separated from the IgY sample named yolkin, were pooled, dialyzed against water, and lyophilized.

### MTT Reduction Assay

Cell viability was determined by colorimetric MTT assay [31]. Cells were seeded onto flat-bottomed 96-well culture plate (1 × 10^4^ cells/well) and incubated for 24 h with yolkin preparation (1–100 µg/ml) at 37 °C for PC12 and 33 °C for H19-7 in a humidified atmosphere of 5% CO_2_ and 95% air for 24 h. After cell treatment, an MTT solution (5 mg/ml) was added, and the cells were incubated again for 4 h to develop formazan crystals. The formazan crystals were solubilized by adding 100 µl DMSO and vigorously shaken to complete resolving. The absorbance was measured at 570 nm by an EnSpire™ 2300 microplate reader (PerkinElmer, MA, USA). Cell viability was expressed as a percentage of non-treated control cells. The experiment was performed at least three times, and each of the samples was triplicated on the plate for technical repetition.

### Western Blot

Yolkin-treated cells and non-treated control cells were lysed in RIPA buffer (150 mM NaCl; 50 mM Tris–HCl, pH 7.5; 5 mM EDTA; 1% Triton X-100; 0.1% SDS; and 0.5% deoxycholate) supplemented with protease and phosphatase inhibitor cocktails (Roche) and 1 mM NaF, on ice for 30 min. Lysates were centrifuged at 12,000 × g for 10 min at 4 °C, and then protein content was determined by the bicinchoninic acid assay using BSA as a standard. Thirty micrograms of protein samples mixed thoroughly with 4 × Laemmli buffer were heated at 95 °C for 5 min, centrifuged, and then separated on 4–12% sodium dodecyl sulfate-(SDS) polyacrylamide gel. Next, the samples were transferred onto 0.44 µm nitrocellulose membranes. Membranes were blocked with 5% nonfat dry milk in Tris-buffered saline with 0.5% Tween 20 (TBST) for 1 h at room temperature. Afterwards, membranes were washed with TBST and then probed overnight at 4 °C with the primary antibodies: anti-ERK 1/2, anti-phospho-ERK1/2, anti-Akt, anti-phospho-Akt, anti-CREB, anti-phospho-CREB, anti-NF-қBp65, anti-phospho-NF-қBp65, anti-CPE, anti-BDNF, and anti-β-actin (all antibodies were used in dilution 1:1000). Next, membranes were probed with secondary antibodies conjugated with alkaline phosphatase (1:10,000) for 1 h at room temperature. Immunocomplexes were visualized using NBT/BCIP substrates, scanned in the Molecular Imager ChemiDoc XRS + Imaging System (Bio-Rad, CA, USA), and analyzed in Image Lab Software. The experiment was performed at least three times.

### Cyclic cAMP Determination by ELISA Assay

Intracellular concentration of cAMP was determined according to the method of Kobiałka et al. [32]. Cells in OPTI-MEM/GlutaMAX medium were transferred into a 48-well plate coated with poly-L-lysine and allowed to rest for 24 h at 37 °C for PC12 and 33 °C for H19-7. Each well contained 200,000 cells in a final volume of 0.6 ml. The next day, cells were left untreated in the control groups or treated with adenosine and used as a positive control (25 µM) or yolkin (100 μg). All samples were prepared in triplicate. After 30 min (adenosine) and 15, 30, and 60 min (yolkin) of incubation, cells were lysed, and intracellular concentration of cAMP was determined. Briefly, the accumulated cAMP was measured using immunoenzymatic assay based on rabbit polyclonal antibodies highly specific for cAMP. To improve sensitivity, the samples and standard solutions of cyclic nucleotides were acetylated before the assay. After incubation with primary antibodies, the samples were washed and incubated for 1 h at room temperature with anti-rabbit IgG goat antibodies conjugated to horseradish peroxidase. The color reaction was developed using tetramethylbenzidine as a substrate, and the absorbance was measured at 450 nm in a Dynatech MR5000 plate reader. The amount of a given cyclic nucleotide in the sample was calculated from a calibration curve prepared for each plate separately. Finally, the results of measurements were expressed as a percentage change in the total cAMP levels in cell populations following treatment relative to the total cAMP level in the untreated cell population of the control group. The experiment was performed four times, and each of the samples was triplicated on the plate for technical repetition.

### Measurement of BDNF Secretion by ELISA Assay

Cells (1 × 10^6^/ml) were suspended in serum-free DMEM medium and plated in 6-well culture plates. Yolkin was applied to the cells and incubated for 2, 6, and 24 h for PC12 cells (100 µg/ml) at 37 °C and for 6, 24, and 48 h for H19-7 (10 and 100 µg/ml) at 33 °C to induce BDNF production. At the end of treatment, supernatants were collected and stored at − 80 °C. Mature BDNF level was measured using a sensitive two-side ELISA kit (BDNF Emax ImmunoAssay System, Promega Corporation, USA) following the manufacturer’s instructions. The experiment was performed at least three times, and each of the samples was duplicated on the plate for technical repetition.

### Measurement of Intracellular Ca^2+^ Concentration

Intracellular level of calcium ions was assessed with Fluo-4 Direct Calcium Assay Kits (Invitrogen). The cells (2 × 10^5^) were resuspended in 100 µl of reagent buffer (125 mM NaCl, 5 mM KCl, 1.2 mM KH_2_PO_4_, 1.2 mM MgSO_4_, 6 mM glucose, 25 mM Hepes (pH 7.5)) with or without calcium (1.2 mM CaCl_2_) and 100 µl of buffer attached to the kit with fluorescent dye Fluo-4 AM. To inhibit the flow of the dye from the cell, the Probenecid (5 mM) was added to the buffer, as well. The samples were incubated at 37 °C for 30 min in the dark. Next, 100 µl of the sample was applied onto a black 96-well plate, and the intensity of the fluorescence was measured (494-nm excitation and 516-nm emission). For the first 20 min, the baseline was measured (unstimulated cells) until it stabilized. Next, the cells were stimulated with yolkin (100 µg/ml or 200 µg/ml), and the fluorescence was measured in the manner of time (60–90 min). For the maximum signal of fluorescence, the cells were treated with ionomycin (5 µM), which released calcium ions from all intracellular resources. The minimal signal was obtained with EDTA addition (50 mM), which chelated all the free calcium ions. The fluorescence intensity changes in time were measured by plate reader Synergy H4 (BioTek Instruments, Winooski, VT, USA). The experiment was performed at least three times, and each of the samples was triplicated on the plate for technical repetition.

### Measure of Nitric Oxide Level

Cells were seeded onto a 48-well plate at a density of 1 × 10^6^/ml, in Dulbecco’s modified medium for cell culture. Yolkin (10 and 100 µg/ml) was added to the cells as a potential inducer of NO. Untreated cells were used as a negative control. After 24 h of incubation, supernatants were collected and the level of NO was determined using a colorimetric method with the Griess reagent [33]. In brief, 100 µl of samples of cell supernatants were incubated with an adequate amount of the Griess reagent (0.1% N-(1-naphthyl)-ethylenediamine and 1% sulfanilamide in 5% phosphoric acid). After 10 min of incubation at room temperature, the absorbance at 550 nm was measured by an EnSpire™ 2300 microplate reader (PerkinElmer, MA, USA). The levels of nitrite were extrapolated using the sodium nitrite standard curve. The experiment was performed at least three times, and each of the samples was triplicated on the plate for technical repetition.

### Data Analysis and Graphical Visualization

Statistics and graphs were prepared using GraphPad Prism Software v9 (San Diego, CA, USA) Data are presented as median ± quartiles (25–75%) and min–max or mean ± SD. Data were analyzed using one-sample *t*-test. A value of *p* < 0.05 or less is considered statistically significant.

## Results

### Yolkin Increased Cell Viability

**Fig. 1:**
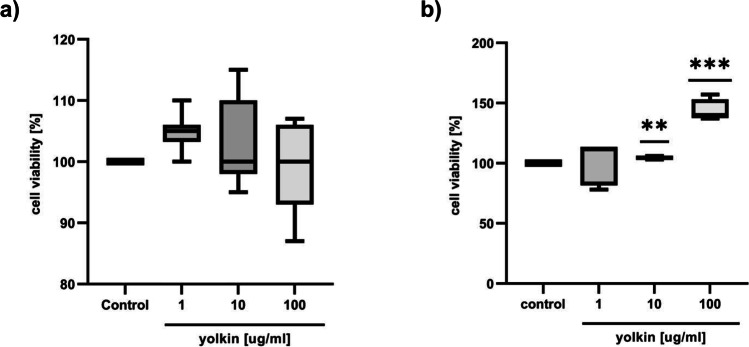
Effect of yolkin on PC12 (**a**) and H19-7 (**b**) viability after 24 h of exposure using MTT assays. PC12 or H19-7 cells (1 × 10^5^/ml) were exposed to yolkin (1, 10, and 100 µg/ml) for 24 h. Cell viability was evaluated by MTT assay. The data are means with min to max values of 4–9 independent experiments. **p < 0.01, *** *p* < 0.001 vs control

We used the MTT assay to monitor the viability of yolkin-treated neural cells. After 24-h incubation with yolkin, a significant increase in the number of viable cells was observed in H19-7 cells as compared to control untreated cells, reaching 104.5% for 10 µg/ml of yolkin and 144.2% for 100 µg/ml of yolkin (Fig. 1b). A discreet but insignificant effect was observed in PC12 cells only for 1 µg/ml of yolkin; however, no effects were seen for 10 and 100 µg/ml of yolkin (Fig. 1a).

### Yolkin Stimulated CREB Phosphorylation at Ser^133^ Residue, but Did Not Activate NF-қB

**Fig. 2:**
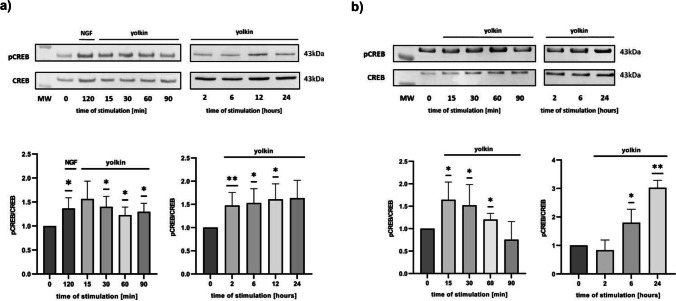
Effect of yolkin on CREB phosphorylation. Ser^133^-phosphorylated form of CREB was detected by Western blotting. Total proteins were extracted from PC12 cells (**a**) and H19-7 cells (**b**) treated with yolkin at concentration of 100 µg/mL, in the time from 15 min to 24 h. Immunocomplexes were visualized in the Molecular Imager ChemiDoc XRS + Imaging System (Bio-Rad) and analyzed in Image Lab Software. The data are means ± S.D. of 3–6 independent experiments. *p < 0.05; **p < 0.01 vs control

Transcription factors CREB and NF-қB participate in the regulation of expression of genes coding pro-survival proteins and neurotrophins, as BDNF [34, 35]. To determine the impact of yolkin on activation of transcription factors CREB and NF-қB, Western blotting analysis were performed using monoclonal antibody specific to Ser^133^-phosphorylated form of CREB and S^536^-phosphorylated p65 subunit of NF-қB factor. During treatment of neuron-like PC12 cells with yolkin, Ser^133^ residue in CREB protein was phosphorylated rapidly, already after 15 min of incubation and was prolonged up to 12 h (Fig. 2a). NGF was used as a positive control. Comparable effect was shown in H19-7 cells; however, two step-activation was visible: fast (started after 15 min of yolkin application and sustained for 60 min) and late (started at 6 h of stimulation and sustained for 24 h) (Fig. 2b). The increase of NF-қB transcription factor activation was not observed after yolkin treatment (data not shown).

### Yolkin Upregulated BDNF and CPE Expression

**Fig. 3:**
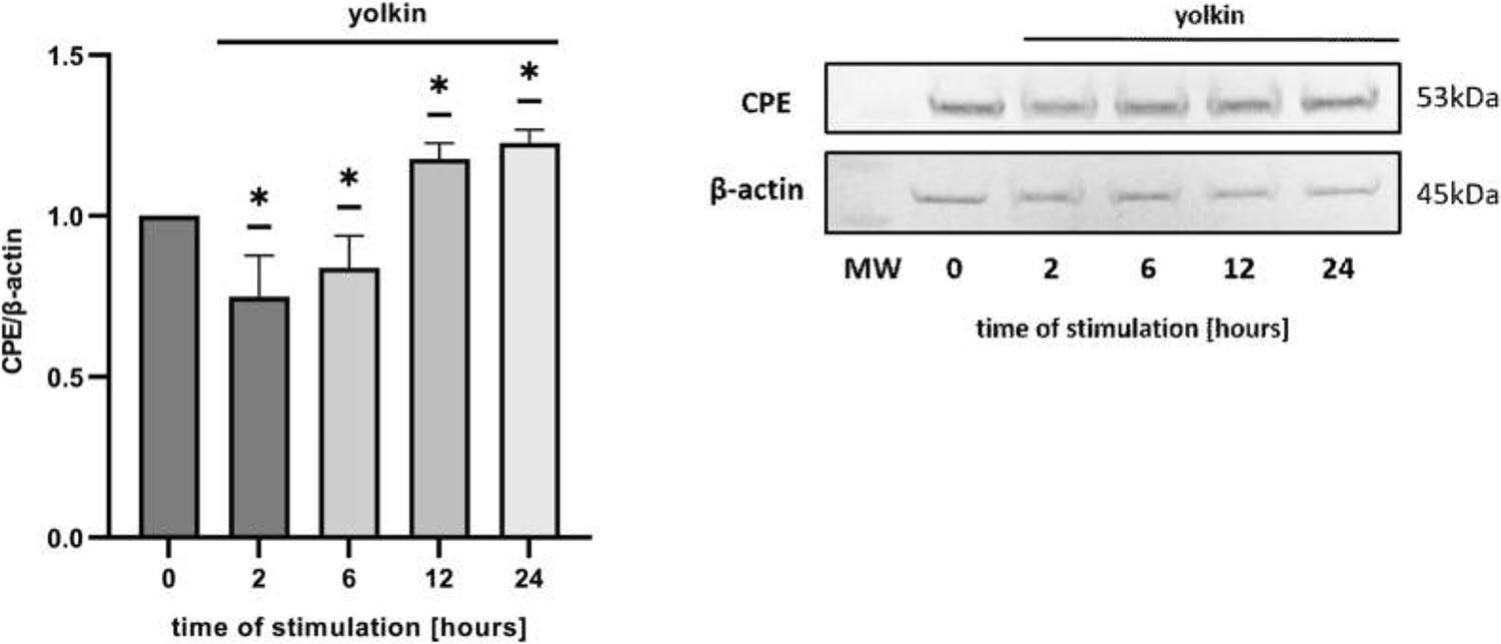
Effect of yolkin on the expression of CPE protein in PC12 cells. PC12 cells (1 × 10.^6^/ml) were treated with yolkin (100 µg/ml) or non-treated (0) for 2–24 h. The expression of CPE protein was determined by Western blot using anti-CPE antibodies; β-actin was used as a loading control. The ratio of CPE/β-actin is normalized by the value in the control group. Values are expressed as the mean ± S.E.M. of three independent experiments. *p < 0.05, versus control group (0)

**Fig. 4:**
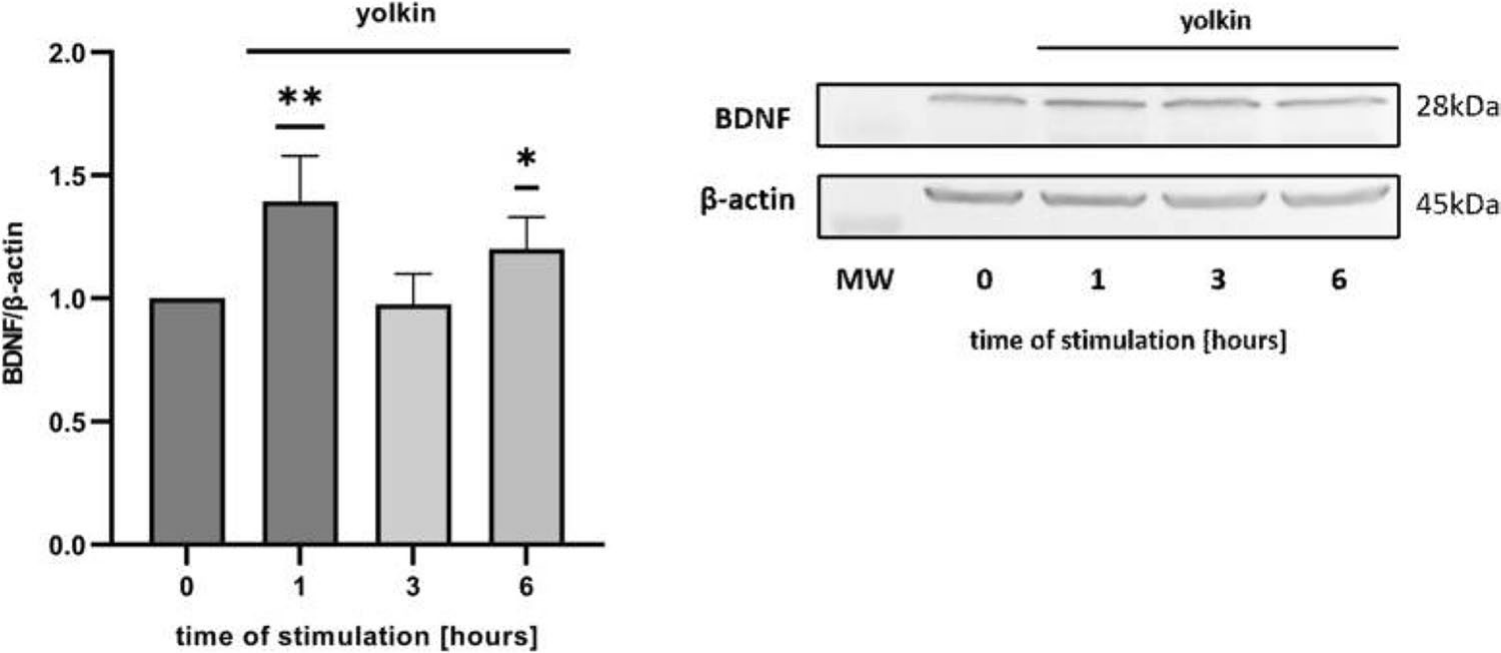
Effect of yolkin on the expression of BDNF protein in PC12 cells. PC12 cells (1 × 10.^6^/ml) were cultured alone (0) or stimulated with yolkin (100 µg/ml) for appropriate time. The expression of BDNF protein was determined by Western blot using anti-BDNF antibodies after 1–6 h of stimulation with yolkin; β-actin was used as a loading control. The ratio of BDNF/β-actin is normalized by the value in the control group. Values are expressed as the mean ± S.E.M (*n* = 3) *p < 0.05, **p < 0.001 versus control group (0)

To determine the changes in BDNF and CPE expression, Western blot analysis was performed. There was a pronounced increase in CPE level, after 12 and 24 h of stimulation with yolkin (100 µg/ml) (Fig. 3). After 1-h treatment of PC12 cells with 100 µg/ml of yolkin, intracellular level of BDNF increased significantly and then decreased a little bit (Fig. 4). Taking together, these results demonstrate that yolkin specifically promotes BDNF and CPE expression in PC12 cells.

### Yolkin Increased BDNF Secretion

**Fig. 5:**
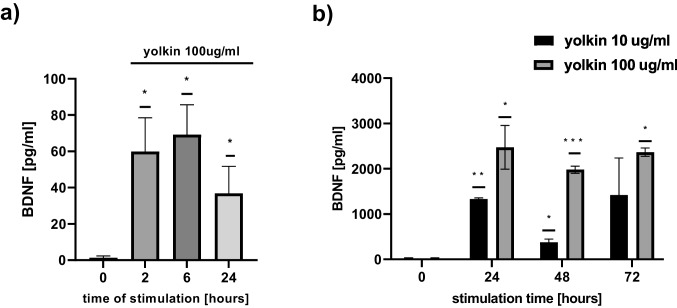
Effect of yolkin on BDNF release from PC12 cells (**a**) and H19-7 (**b**). Yolkin was applied to the cells and incubated for 2, 6, and 24 h for PC12 cells (100 µg/ml) and for 6, 24, and 48 h for H19-7 (10 and 100 µg/ml) to induce BDNF production. Control cells (0) were incubated in the absence of inducers. The concentration of mature BDNF in the cell-free supernatants was determined by ELISA (*n* = 7 for PC12 and *n* = 3 for H19-7). The differences between groups were analyzed using one-sample *t*-test. *p ≤ 0.05 statistically significant differences *vs* control

It was shown that yolkin, in dose-dependent manner, increased the expression and production of mature BDNF in both PC12 (Fig. 5a) and H19-7 (Fig. 5b) cells. Compared to the control group, yolkin increased BDNF level already after 2-h treatment in PC12 cells (*p* ≤ 0.05) and only after 24-h treatment in H19-7 cells (*p* ≤ 0.05). Our data also have shown that in PC12 cells (which are TrkB-deficient cells), BDNF expression falls significantly after 24 h of incubation with yolkin, whereas in H19-7 cells (which are TrkB-positive cells), the level of secreted BDNF slightly increased after 24-h treatment with yolkin and stayed high after 48 h.

### Yolkin-Dependent CREB Phosphorylation Is Upregulated by cAMP/PKA and PI3K/Akt Kinases

**Fig. 6:**
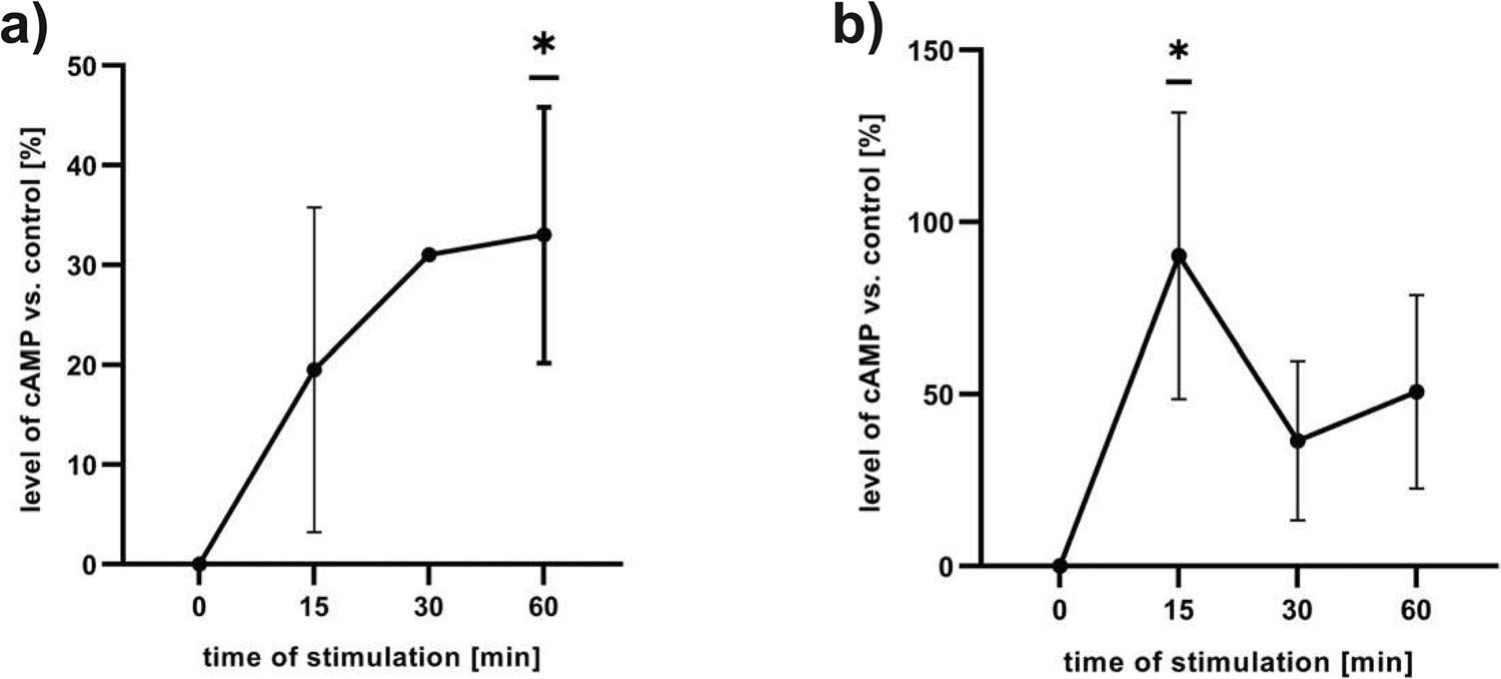
Activation of cAMP production by yolkin-treated PC12 cells (**a**) and H19-7 cells (**b**). PC12 or H19-7 cells were left untreated in the control groups (0) or treated with yolkin (100 µg). All samples were prepared in triplicate. After 15, 30, and 60 min of incubation with yolkin cells were lysed, and intracellular concentration of cAMP was determined by ELISA assay. The data are the mean ± SD (*n* = 4). *p ≤ 0.05, statistically significant difference in the value between the yolkin-treated and non-treated control cells (0)

To identify CREB-related signaling pathways activated by yolkin, Western blotting was performed. Ser^133^ residue of CREB factor can be phosphorylated in response to increase of intracellular level of cAMP, NO/cGMP, or Ca^2+^. When PC12 or H19-7 cells were incubated with yolkin, no changes in intracellular Ca^2+^ level and in secreted nitric oxide level were observed (data not shown). However, a significant increase in intracellular cAMP level was observed. Treatment of both PC12 (Fig. 6a) and H19-7 (Fig. 6b) cell lines with yolkin (100 µg/ml) increased cAMP level in a time-dependent manner. A significant increase in the cAMP level was observed 60 min after the yolkin application to PC12 cells and 15 min after yolkin application to H19-7 cells.

**Fig. 7:**
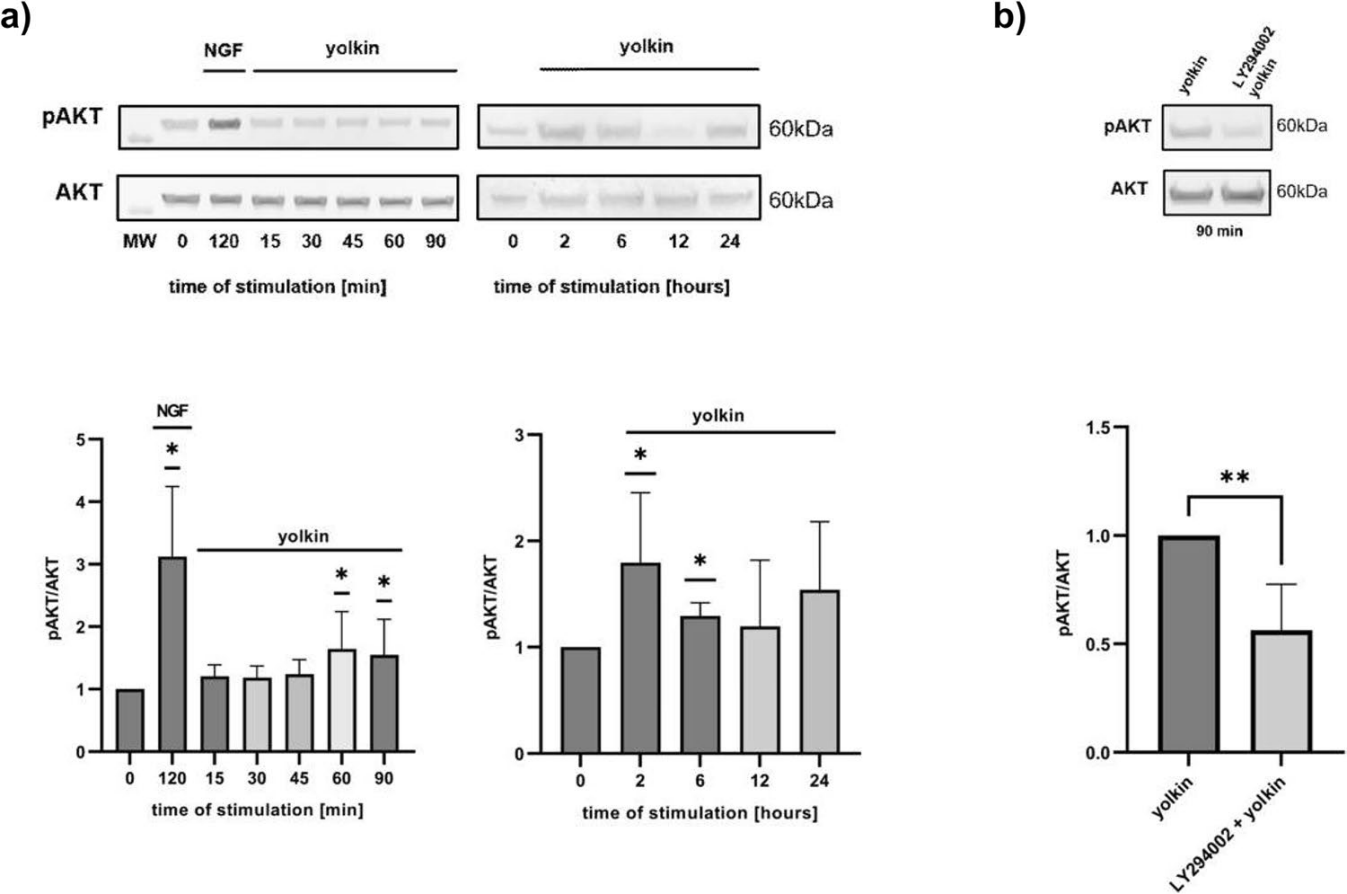
Effect of yolkin treatment on PI3K/Akt kinase activation in PC12 cells. PC12 cells were treated with yolkin (100 µg/ml) for short- and long-term stimulation. Next, cells were lysed and subjected to SDS-PAGE followed by Western blotting using monoclonal anti-Akt or anti-phospho-Akt antibodies. The specific activity of yolkin was assessed by 1-h pre-incubation of cells with LY294002, a selective PI3K inhibitor and next with yolkin for 90 min (**b**). Data were expressed as a fold of change in density compared with control cells (0). Immunocomplexes were visualized in the Molecular Imager ChemiDoc XRS + Imaging System and analyzed in Image Lab Software. All experiments were repeated three times. The data are means ± S.D. *p < 0.05; **p < 0.01 vs control

**Fig. 8:**
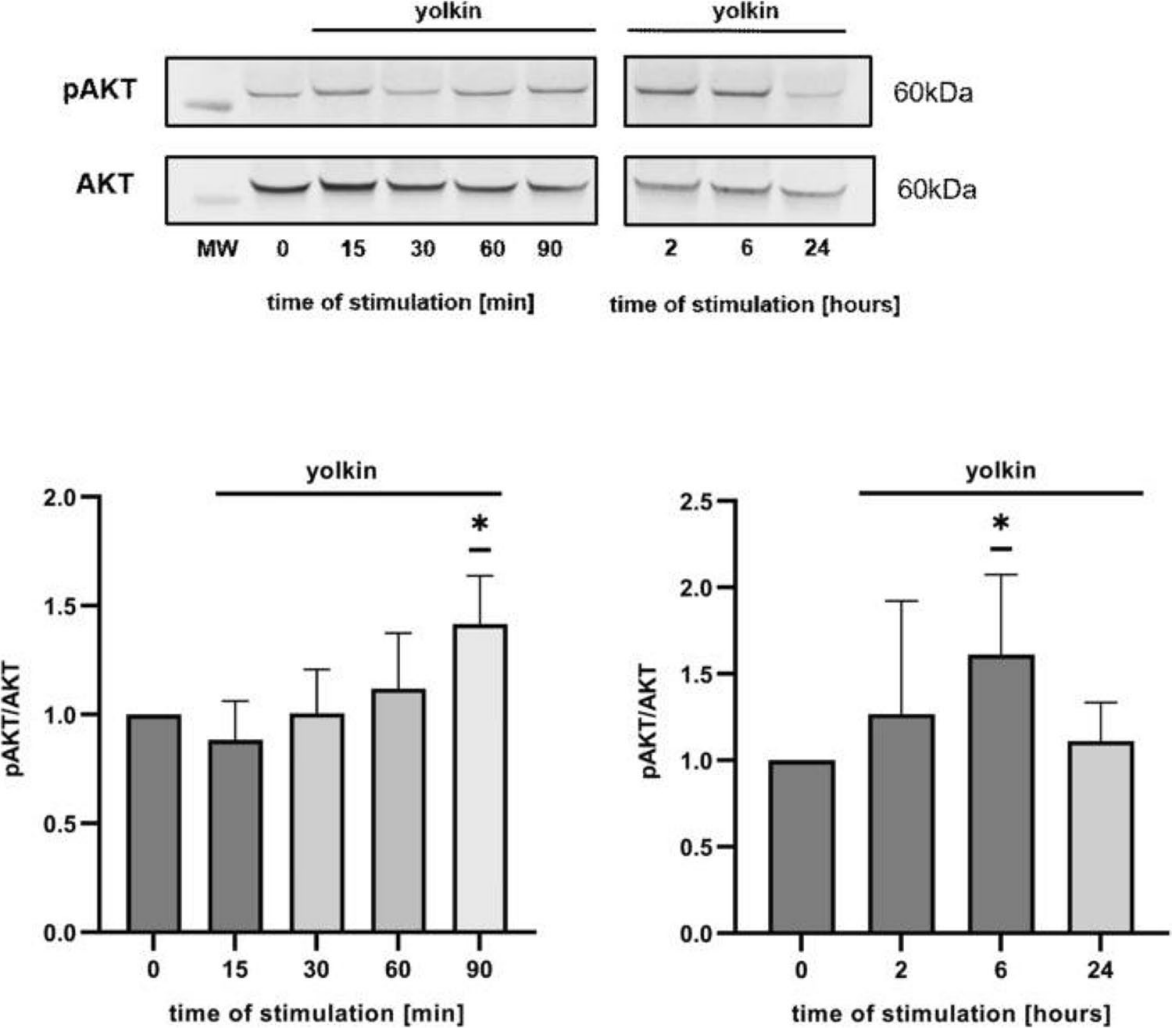
Effect of yolkin treatment on PI3K/Akt kinase activation in H19-7 cells. H19-7 cells were treated with yolkin (100 µg/ml) for short- and long-term stimulation. Next, the cells were lysed and subjected to SDS-PAGE followed by Western blotting using monoclonal antibody anti-Akt or anti-phospho-Akt. Data were expressed as a fold of change in density compared with control cells (0). Immunocomplexes were visualized in the Molecular Imager ChemiDoc XRS + Imaging System and analyzed in Image Lab Software. All experiments were repeated three times. The data are means ± S.D. *p < 0.05 vs control

Given that ERK 1/2 and PI3K/Akt kinases phosphorylate CREB, we assessed the impact of yolkin on their activation. Relatively, no effect was found on ERK1/2 kinase phosphorylation (data not shown), whereas it was found that yolkin significantly increased the phosphorylation level of Akt in PC12 cells (Fig. 7a) after 60 min of incubation until 6 h. Moreover, LY294002, a selective PI3K inhibitor, inhibited yolkin-induced Akt phosphorylation in PC12 cells (Fig. 7b). When H19-7 cells were incubated with yolkin, significant increase of Akt phosphorylation level was observed after 90 min and lasted until 6 h (Fig. 8).

## Discussion

In the present study, the molecular mechanisms activated by yolkin complex in PC12 and H19-7 cell lines were examined. Our findings demonstrate that yolkin, due to the activation of cAMP/PKA and PI3K/Akt pathways, can upregulate CREB phosphorylation and BDNF expression.

Cognitive function decline is strictly related to age. Aging can lead to brain dysfunction including hippocampal neurons degeneration or cognitive and mood disorders [2]. Hippocampal neurons express high levels of the neurotrophins like nerve growth factor (NGF), BDNF, and CPE/NF-α1 [12, 13]. It was demonstrated that disturbance of neuronal plasticity, memory, and cognition, observed during aging, resulted in the decreased level of released neurotrophins and reduced expression of Trk receptors on the cell surface [36–38]. It has been also reported that induction of BDNF production/secretion in the hippocampus of aged mice could rescue the long-term potentiation and relieve spatial memory deficits [5, 39].

It has been proven that an adequate diet and balanced supplementation may significantly inhibit neurodegenerative processes improving cognitive functions [19, 20, 40, 41]. Therefore, intense research has been conducted on cognitive-enhancing treatment, especially with substances of natural origin. One of them could be yolkin polypeptide complex isolated from hen egg yolk. Behavioral studies performed previously by Lemieszewska et al. [28] showed the beneficial impact of yolkin on cognitive functions in young and old rats and used as a model of cognitive decline and processes of the brain aging. Yolkin, applied orally, mitigated behavioral symptoms of aging and supported cognitive learning and memory in rats from both age groups. We also observed that yolkin, in dose-dependent manner, stimulate PC12 cells to produce and secrete significant amounts of mature BDNF [42]. This was also observed in the human whole blood ex vivo [43]. To understand the molecular basis of yolkin activity, two cellular models, rat pheochromocytoma PC12 cell line, which is TrkB-deficient, and immortalized rat hippocampal precursor cells H19-7, which expresses TrkB receptor, were used [29, 30, 44–46]. The lack of the TrkB receptor in PC12 cells exclude the possibility of its activation by BDNF secreted to the supernatant in response to yolkin. Both cell lines have several features to measure differentiation, neuroprotection, and neurosecretory activity.

It is well-known that CREB is a key transcriptional regulator, which can be activated by a variety of external signals and regulates the transcription of neurotropic factors and synaptic and pro-survival proteins [15, 47, 48]. During an early developmental stage, CREB controls vital neuronal functions like cell proliferation, differentiation, growth, and survival. However, in the adult brain, it plays an important role in synaptic plasticity, learning, and memory formations [49]. As shown by MTT assay, in the presence of yolkin, survival rate and proliferation of neuronal cells are promoted. After the treatment of PC12 cells with yolkin, cell viability was unchanged compared to the untreated control cells (Fig. 1a). Interestingly, after treatment of hippocampal precursor cells H19-7 (TrkB +) with yolkin, a significant increase of proliferative activity was observed (*p* ≤ 0.05). Simultaneously, we also observed that yolkin upregulated expression/production of BDNF in both PC12 and H19-7 cells. These results may suggest that the presence of the TrkB receptor is not necessary for CREB-dependent BDNF expression induced by yolkin observed in PC12 cells. We can also speculate that increased prosurvival activity of hippocampal precursor cells H19-7 treated with yolkin can be a result of yolkin-stimulated BDNF production, which released to intercellular space binds to TrkB receptor and trigger CREB-dependent pathway responsible for control of H19-7 cells proliferation (Fig. 1b) [50]. Taking into account the role of hippocampal neurogenesis, the effect of yolkin on hippocampal precursor cells H19-7 proliferation may be of importance to neuronal development and survival, especially in prevention of neurodegenerative diseases.

Hippocampal neurons express high levels of CPE which acts as a sorting receptor for targeting pro-BDNF to the regulated secretory pathway for processing [12, 13]. It is also involved in secretory granule transport to the plasma membrane by interaction with dynactin and kinesin microtubule motors [51]. Knock-out mice lacking CPE/NF-α1 exhibit some neurological defects including learning and memory deficits [14, 52]. In turn, Xiao et al. provided evidence that the lack of CPE had a major effect on rendering the BDNF-TrkB system non-functional in CPE-KO mice [13]. The latest data of Sharma et al. demonstrated that CPE promotes neuroprotection of human neurons during induced oxidative and neuro-excitotoxic stress by activation of β-arrestin/ERK/CREB/Bcl2 pathway [53]. Based on these reports and due to the observed significant impact of yolkin on BDNF expression, it seemed reasonable to investigate the effect of yolkin on the CPE expression. We have discovered, for the first time, that yolkin upregulates the expression of CPE protein in PC12 cells (Fig. 3). RT-qPCR analysis showed an increased level of *cpe* mRNA, however, insignificant (data not shown). In turn, Western blotting results presented a significant decrease of intracellular CPE level up to 6 h of incubation of PC12 cells with yolkin. It is correlated with significant upregulation of mature BDNF secretion to the supernatant observed already after 2 h of incubation with yolkin with maximal secretion observed after 6 h (Fig. 5a). Subsequently, a significant increase of intracellular CPE expression was started after 12 h of incubation with yolkin, which is likely to correlate with an increase in BDNF expression, and it is probably processing. This is the first published results showing that a naturally derived preparation yolkin can enhance the expression of two interacting neurotrophic factors: BDNF and CPE, playing a crucial role in the control of survival and functioning of the central nervous system neurons.

To understand the mechanisms involved in the neuroprotective activity of yolkin, the study focused on explaining which signaling pathways responsible for CREB phosphorylation and BDNF expression are activated in yolkin-treated cells. The crucial event in the activation of CREB is phosphorylation of Ser133 which can be triggered by a variety of signaling processes including an increase in intracellular Ca^2+^ level [54], NO/sGC (soluble guanylyl cyclase)/cGMP pathway[55], an increase in cAMP and PKA activation [56], and activation of ERKs [56] or PI3K/Akt [57]. All pathways directly activate CREB transcription factor [15, 58]. Firstly, the impact of yolkin on intracellular Ca^2+^ level was determined. Unfortunately, no changes in intracellular Ca^2+^ level in response to yolkin stimulation were observed both in PC12 and H19-7 cells (data not shown). There were also no changes in the level of secreted nitric oxide (data not shown). Moreover, we found out that yolkin-dependent CREB phosphorylation does not require activation of ERK 1/2 kinases (data not shown). Interestingly, as it was shown in Fig. 6, a significant upregulation of cAMP level in yolkin-treated PC12 and H19-7 cells were observed (*p* ≤ 0.05). It may suggest that yolkin can promote cAMP-dependent activation of PKA, and subsequently PKA is able to activate CREB by Ser^133^ residue phosphorylation, thereby further mediating BDNF expression. Comparable activity was observed to curcumin [59].

Additionally, we assessed the impact of yolkin on PI3K/Akt activity. When PC12 cells were treated with 100 µg/ml of yolkin preparation, the level of Akt phosphorylation was increased significantly (*p* ≤ 0.05) (Fig. 7a). As a specificity control of yolkin action 1-h pre-incubation of cells with LY294002, a selective PI3K inhibitor was performed, which confirmed yolkin Akt-dependent phosphorylation (Fig. 7b). The ability of yolkin to activate the Akt kinase was also confirmed in H19-7 cells (Fig. 8).

Summarizing, this is the very first attempt to clarify the intracellular signaling pathways activated by yolkin in neural cells. Our work explains the molecular basis of the biological activity of the egg yolk–derived yolkin complex, which is mediated by PI3K/Akt and cAMP/PKA signaling pathways, leading to upregulation of CREB phosphorylation and increased expression of BDNF and CPE, important neurotrophic factors. We also suspect that the presence of the TrkB receptor is not necessary to yolkin-dependent CREB phosphorylation and BDNF expression, which is observed in PC12 (TrkB(-)) cells. Our results, demonstrating neurotrophic potential of yolkin, are consistent with previous reports, showing similar neuroprotective roles exerted by other natural substances like genistein and daidzein [50]. Also polyphenols such as epigallocatechin gallate, curcumin, epicatechin, quercetin, resveratrol, or citrus flavonoids can interfere directly with intracellular signaling molecules to alter brain activity [60–62].

## Conclusion

It is very important to establish an effective way to enhance healthy aging and delay age-related diseases. There is relevant evidence confirming an association between healthy diet and cognitive functions. The current knowledge indicates that the proper enrichment of diet can reduce the risk of cognitive deficits. There is still a lack of effective pharmaceutical treatments, helping to age healthily. The cellular mechanisms underlying the neuroprotective activity of nutraceuticals must be elucidated to uncover a novel approach for developing drugs. Our studies have explained the potential molecular mechanisms of activity of egg yolk–derived complex, which is mediated by PI3K/Akt and cAMP/PKA signaling pathways, leading to upregulation of CREB phosphorylation (but not NF-қB), and increased expression of BDNF and also CPE—an important neurotrophic factors. It was also shown that the presence of the TrkB receptor is not necessary to yolkin-dependent CREB phosphorylation and BDNF expression. These results emphasize that yolkin can act comprehensively and in many directions, causing a beneficial effect on neurons survival and activity. Therefore, it seems that yolkin specimen can be used as a safe, bioavailable, natural nutraceutical, improving memory and learning processes in older people.

## Data Availability

The datasets generated during current study are available from the corresponding author on the reasonable request.

## Abbreviations

IgY: Immunoglobulin Y
BDNF: Brain-derived neurotrophic factor
cAMP: Cyclic adenosine monophosphate
CREB: Cyclic adenosine monophosphate–response element-binding protein
CPE/(NF-α1): Carboxypeptidase E/neurotrophic factor–α1
PKA: Protein kinase A
ERK 1/2: Extracellular signal-regulated kinases 1/2
PI3K/Akt: Phosphoinositide 3-kinases/protein kinase B
NF-қB: Nuclear factor қB
CNS: Central nervous system
MAPK: Mitogen-activated protein kinases
TrkB: Tropomyosin receptor kinase B
Aβ: Amyloid beta
Bcl-2: B-cell lymphoma 2
MnSOD: Manganese superoxide dismutase
NMDA receptor: N-methyl-D-aspartate receptor
GPC receptors: G-protein-coupled receptors
CAMKII/IV: Ca2 + /calmodulin-dependent protein kinase II/IV
NO: Nitric oxide
cGMP: Cyclic guanosine monophosphate
NGF: Nerve growth factor
